# Periodontal Indices and Cytokines of Ex-Smokers/Non-Smokers After Bleaching

**DOI:** 10.3390/medicina62091818

**Published:** 2026-09-21

**Authors:** Mehmet Durdu, Derya Gursel Surmelioglu, Gorkem Kervancioglu, Merve Yilmaz, Zeyneb Merve Özdemir, Hasan Ulusal

**Affiliations:** 1 İstanbul Oral and Dental Health Center, Istanbul 34218, Türkiye; 2 Department of Restorative Dentistry, Faculty of Dentistry, Gaziantep University, Gaziantep 27310, Türkiye; 3 Department of Restorative Dentistry, Faculty of Dentistry, Ondokuz Mayıs University, Samsun 55270, Türkiye; 4 Department of Restorative Dentistry, Faculty of Dentistry, Kahramanmaras Sutcu Imam University, Kahramanmaras 46040, Türkiye; 5 Department of Medical Biochemistry, Faculty of Medicine, Gaziantep Islam Science and Technology University, Gaziantep 27010, Türkiye

**Keywords:** cytokines, ex-smokers, non-smokers, periodontal index, tooth bleaching

## Abstract

*Background and Objectives:* Smoking is known to modulate the periodontal inflammatory response, and dental bleaching agents can transiently alter local and systemic cytokine levels. The aim of this study was to evaluate cytokine levels in both saliva and gingival crevicular fluid (GCF) in non-smoking and ex-smoking patients, before and after in-office dental bleaching performed using chemically activated or diode-laser-activated protocols. *Materials and Methods:* This prospective, longitudinal, split-mouth clinical study included 40 participants allocated to two smoking-status groups (non-smokers and ex-smokers, cessation ≥ 5 years). For each participant, one maxillary hemi-arch received chemically activated bleaching and the contralateral side received diode-laser-activated bleaching, applied in two sessions one week apart. Plaque index (PI), gingival index (GI), and bleeding index (BI) were recorded, and IL-6 and TNF-α levels were measured in saliva and GCF using ELISA at baseline, after each session, and two weeks after treatment. *Results:* A non-significant increase was observed in GI and BI after two weeks compared with baseline (*p* > 0.05), while a significant decrease was observed in PI in the ex-smoker group (*p* < 0.05). A transient increase in TNF-α and IL-6 levels was observed in saliva and GCF samples following bleaching, and TNF-α/IL-6 levels were consistently lower in the ex-smoker group across all measurements. *Conclusions:* Bleaching was associated with a temporary alteration of periodontal indices and cytokine levels irrespective of smoking history. Smoking history was independently associated with lower cytokine levels, regardless of bleaching application, supporting the relevance of considering smoking history when interpreting the inflammatory impact of bleaching activation methods.

## 1. Introduction

Bleaching is a process that transforms dark-colored, high-molecular-weight, double-carbon bonded compounds into light-colored, low-molecular-weight, single-carbon compounds. This process often employs hydrogen peroxide (HP) and its derivatives as the primary agents for bleaching treatment [1]. HP, being an unstable molecule, can generate a significant quantity of free radicals. Free radicals are molecules that possess one or more unpaired electrons in their molecular or atomic structure, rendering them capable of easily exchanging electrons with other molecules. They are also referred to as reactive oxygen species (ROS) or oxidant molecules [2]. Substances that are capable of neutralizing or preventing the harmful effects of oxidant molecules within the body, or that can delay their oxidation, are referred to as antioxidants. Antioxidants, which are naturally present in the body, serve to limit the activity of oxidant molecules. Oxidative stress may lead to the damage of epithelial cells by causing premalignant transformations [3]. Additionally, HP has been demonstrated to have scientific validity and is commonly utilized in appropriate concentrations. Nevertheless, when utilized in high concentrations (>35%), it can result in serious toxic effects, particularly when ingested or when it comes into contact with the eyes [4].

Clinical bleaching agents typically comprise various concentrations of HP, and are activated by the application of heat, light, or chemical activation methods, which also accelerate the reaction [5]. A study suggests that a temperature rise of 5.5 °C caused by heat activation can have detrimental consequences for the pulp [6]. To mitigate these potential side effects, the use of alternative light sources and chemical catalysts for activation is generally considered a safer approach [7]. The addition of pigments to the gel is intended to enhance the absorption of the activating light [8]. One of the most frequently utilized devices, known as a diode laser, is often preferred due to its ease of use, lightness and portability, and relatively brief application time [9]. Dental lasers are designed with specialized tips for use in teeth whitening treatments. These tips spread the incident beam more widely, thereby increasing the affected surface area, which allows for quicker treatment completion and reduces the risk of tissue damage [6].

Cytokines are low-molecular-weight glycoproteins that hold significant importance in various biological processes, including cell growth, inflammation, immunity, tissue repair, and hematopoiesis. Additionally, they play a critical role in the inflammatory response associated with gingivitis, tissue destruction in periodontal diseases, and the regulation of the adaptive immune response [10]. Experimental tooth bleaching has been shown to induce cytotoxicity and pain conduction in dental pulp stem cells via intracellular ROS generation [11]. An earlier study found that bleaching gels suppressed dental pulp stem cell viability and increased intracellular ROS levels [12]. Furthermore, the mRNA and protein expression of IL-6 and TNF-α were upregulated both in vitro and in vivo following bleaching treatment [11]. The levels of certain cytokines, including interleukin-1-β (IL-1-β), interleukin-6 (IL-6), and tumor necrosis factor alpha (TNF-α), are known to fluctuate in response to various stimuli. During the bleaching treatment, inflammation may be induced in the pulp–periodontium complex due to the adverse effects of HP and the increase in pulp temperature resulting from the application of heat and light sources.

The aim of this study was to evaluate cytokine levels in both saliva and gingival crevicular fluid (GCF) in non-smoking and ex-smoking patients, before and after in-office dental bleaching performed using chemically activated or diode-laser-activated protocols.

The primary aim of this study was to evaluate whether the temporal course of clinical and biochemical responses to in-office dental bleaching, and specifically the interaction between smoking history and activation method (laser-activated vs. non-activated bleaching) over time, differs between non-smokers and ex-smokers. We hypothesized that the effect of activation method on tooth color change and on gingival crevicular fluid (GCF) inflammatory markers would vary according to smoking status, and that this smoking status × activation method interaction would itself change across the three assessment time points (baseline, immediately post-bleaching, and follow-up).

## 2. Materials and Methods

Ethics approval was granted by the Local Ethics Committee of the Gaziantep University Faculty of Dentistry, as evidenced by the decision number 2020/415, and was conducted in accordance with the Helsinki Declaration. The study was conducted over a one-year period within the Department of Restorative Dentistry at the Faculty of Dentistry, Gaziantep University.

### 2.1. Sample Size

Sample size was estimated a priori using G*Power 3.1.9.4 based on the mean difference in the primary outcome reported by comparable published bleaching/GCF studies, an expected within-subject correlation of r = 0.50 between the two split-mouth sides, α = 0.05, and 80% power, which indicated a minimum of 20 participants per smoking-status group. A post hoc sensitivity analysis confirmed that, with the achieved sample of 40 participants (approximately 10 per smoking status × activation method combination), the study had adequate power (≥80%) to detect the main effects of activation method and time and their two-way interaction; however, power for the three-way interaction (smoking status × activation method × time) was more limited, and results for this specific interaction should be interpreted as exploratory. This is acknowledged as a limitation in Section 4.

#### Inclusion and Exclusion Criteria

Included were patients with no comorbidity or medical prescription for any kind of systemic disease, aged between 18 and 40 years of age, with time available to participate in the research, with no restored maxillary or mandibular anterior teeth, with absence of tooth sensitivity (stimulated sensitivity with air syringe), and with tooth color A2 to A3.5 according to the Vita Classic Shade Guide. Periodontal health status was determined through an evaluation of periodontal condition based on the criteria established by the World Health Organization in 1982 [13]. Measurements were conducted using the WHO probe [14]. The state of gingiva for the groups was healthy, and the mean of GI was less than 1. Excluded were individuals having both periodontitis and gingivitis.

### 2.2. Participants (Smoking-Status Classification)

Participants were allocated to one of two smoking-status groups based on a structured interview and self-report validated at inclusion: non-smokers (never smokers) and ex-smokers (former smokers who had ceased smoking for at least 12 months prior to enrolment; in the present cohort, all ex-smokers had a cessation history of more than 5 years). No current/active smokers were enrolled in this study. These two group labels are used consistently throughout the manuscript, tables, and figures; the terms “smoker” and “ex-smoker” are not used interchangeably.

### 2.3. Study Design

This prospective, longitudinal, randomized split-mouth clinical study included two bleaching sessions performed one week apart. Periodontal indices were evaluated at baseline, after the second bleaching session, and two weeks after treatment. Saliva and GCF cytokine levels were assessed at four time points: baseline, after the first bleaching session, after the second bleaching session, and two weeks after the final session. For each participant, the two maxillary hemi-arches (right and left) were randomly assigned, using a computer-generated randomization sequence, to receive either laser-activated or non-activated (chemical-only) bleaching, so that each participant served as their own control. The maxillary central incisors, lateral incisors, and canines (teeth 13–23) were the only teeth treated and evaluated; mandibular teeth were not included. Periodontal parameters (probing depth, bleeding on probing, plaque index) and GCF sampling were performed only at the treated maxillary anterior teeth on each side, not across the entire dentition. The split-mouth design was employed in accordance with previously validated protocols for intra-subject comparison of dental bleaching procedures [15].

The subjects in the study were thoroughly briefed about the application and were required to provide an informed consent form that was duly signed. The attending physician verbally communicated the importance of ceasing smoking both before and after the bleaching procedure to all patients, and this information was also conveyed in written form. The ex-smokers group comprises individuals who had previously engaged in long-term smoking and ceased the habit for five years or more.

Forty patients were divided into two smoking-status groups (non-smokers/ex-smokers). For each participant, the two maxillary hemi-arches were randomly assigned to receive chemical or diode-laser-activated bleaching, so that every participant contributed one chemically activated side and one laser-activated side (split-mouth design); the mandibular arch was not treated or evaluated. For descriptive purposes, the four resulting side-by-smoking-status combinations are labeled as follows: Group 1: Non-Smoker/Chemical side; Group 2: Non-Smoker/Laser side; Group 3: Ex-Smoker/Chemical side; Group 4: Ex-Smoker/Laser side.

#### 2.3.1. Bleaching Application

Prior to the administration of the bleaching procedure, patients were captured through photography. The study participants were provided with toothbrushes and toothpaste that contains whitening agents (Colgate Optic White, Colgate-Palmolive Company, New York, NY, USA) so as to ensure uniformity in their dental care routine. The application of a bleaching agent (Whiteness HP Blue Calcium, FGM Dental Products, Joinville, SC, Brazil) comprising a content of 35% HP in a blue-purple tint was carried out in accordance with the manufacturer’s instructions for both groups of patients. The bleaching agent concentration and application protocol followed the parameters established in [16].

The bleaching agent was applied for ten minutes three times and then removed with suction. After the agent was placed on the teeth, the bleaching agent on one randomly assigned maxillary hemi-arch was left undisturbed, whereas the bleaching agent on the contralateral maxillary hemi-arch was activated with a 980 nm diode laser (continuous mode) at an output power of 4 W, with a spot size of 5.85 cm^2^ (corresponding to an energy density of 13.6 J/cm^2^), held at a distance of approximately 5 mm from the tooth surface, using a continuous sweeping motion across the vestibular surface of each tooth, at 5 min intervals, applied for 20 s to each tooth. Diode-laser activation parameters (wavelength, power, spot size, exposure mode) were selected based on previously published, clinically validated protocols [17]. Protective eyewear was worn by the patient and operator throughout laser activation. During the bleaching process, after placement of a rubber dam, a bleaching agent was applied to the half arches that received laser treatment. The total time for the whitening treatment was 30 min, and it was conducted in two sessions with a one-week interval between them.

#### 2.3.2. Oral Hygiene Protocol

All participants received the same commercially available, whitening fluoride toothpaste (Colgate Optic White, Colgate-Palmolive Company, New York, NY, USA; 0.76% sodium monofluorophosphate, silica, and hydrogen peroxide) and a standard soft-bristle toothbrush at enrolment, with instructions to use only this product, twice daily, for the entire study period. Use of any other whitening, desensitizing, or antiseptic oral care product was an exclusion criterion and was reassessed by interview at each visit. As the same product was used identically on both split-mouth sides, its potential confounding effect on the primary side-to-side (activation method) comparison is minimized; nonetheless, a possible non-specific whole-mouth effect on salivary markers cannot be entirely excluded and is discussed as a limitation.

Periodontal index measurements were conducted on patients prior to initiating bleaching treatments. Specifically, the plaque index (PI), gingival index (GI), and bleeding index (BI) were assessed.

#### 2.3.3. Plaque Index (PI) Measurement

The plaque indices were assessed based on the criteria established by Silness and Löe [18].

#### 2.3.4. Gingival Bleeding Index (BI) Measurement

Gingival bleeding was evaluated using the gingival bleeding index (GBI), and bleeding on probing was assessed as presence (1) or absence of bleeding (0) at six sites for each tooth according to Ainamo and Bay [19]. This methodology simplified the analysis by focusing on the presence or absence of gingival bleeding rather than its severity.

The application of the periodontal probe to the gingiva with a moderate level of pressure was performed on the anterior teeth, and the subsequent assessment of bleeding was conducted.

#### 2.3.5. Gingival Index (GI) Measurement

The evaluation assessed the buccal, mesial, distal, and lingual/palatal gingiva of the teeth, and the GI was calculated as the mean of the measurements [20].

#### 2.3.6. Saliva Sampling

Salivary samples were obtained from the subjects at the start of the day (9–12 am) to minimize the influence of diurnal fluctuations on the composition of saliva. Subjects were instructed not to consume food or beverages, use mouthwash, or chew gum within a two-hour period prior to the collection. Unstimulated saliva was collected from participants. The individuals were required to remain seated in silence for 5 min before their saliva was collected, and the samples were collected in plastic tubes for a period of five minutes. The collected saliva samples were then stored at a temperature of −80 °C until analysis. Each saliva sample was stored after centrifugation. Unstimulated whole saliva was collected according to a previously validated protocol [21].

#### 2.3.7. Gingival Crevicular Fluid (GCF) Sampling

In each participant, GCF was sampled from the maxillary canine on each side (tooth 13 and tooth 23), selected a priori as the reference tooth because of its consistent presence, accessibility, and comparable gingival biotype across participants; the same tooth and the same mesio-buccal sampling site were used at every time point (T0, T1, T2) to ensure within-subject comparability. Prior to sampling, supragingival plaque was gently removed with a sterile curette without touching the gingival margin, and the site was isolated with cotton rolls and gently air-dried for 5 s to avoid dilution of the sample by saliva. A standardized filter-paper strip (Periopaper^®^, Oraflow Inc., Smithtown, NY, USA) was inserted into the gingival sulcus/pocket at the mesio-buccal line angle until mild resistance was felt, and left in place for 30 s; strips visibly contaminated with blood were discarded and re-sampling was performed at an adjacent site after a 5 min interval. Collected volume was quantified using a calibrated device (Periotron^®^ 8000, Oraflow Inc., Smithtown, NY, USA), and strips were immediately placed in coded microcentrifuge tubes and stored at −80 °C until analysis. GCF was collected using the filter-paper strip (Periopaper) technique, following methodology previously validated and widely applied in periodontal biomarker research [22,23].

Samples were obtained from patients following the initial session, after the first session, at the subsequent session, and two weeks thereafter for both saliva and GCF. The samples were analyzed for the levels of IL-6 and TNF-α using sandwich enzyme-linked immunosorbent assay (ELISA) kits. IL-6 and TNF-α concentrations in GCF and saliva were measured using commercially available sandwich ELISA kits (IL-6: FineTest, Wuhan, China; catalog no. EH0201; TNF-α: FineTest, Wuhan, China; catalog no. EH0302), according to the manufacturer’s protocols. The assay range was 4.688–300 pg/mL for IL-6 and 15.625–1000 pg/mL for TNF-α, with sensitivity of 2.813 pg/mL and 9.375 pg/mL, respectively. The manufacturer-reported intra-assay and inter-assay coefficients of variation were 4.66% and 4.64% for IL-6, and 5.56% and 5.49% for TNF-α. All samples were run in duplicate on the same microplate where possible, and a standard curve was generated for each plate using the kit’s reference standards. In summary, according to these kit protocols, this kit was based on sandwich enzyme-linked immunosorbent assay technology. Antibody was pre-coated onto the 96-well plate. The biotin conjugated antibody was used as the detection antibody. The standards and samples were added to the wells subsequently. After incubation, unbound conjugates were removed by wash buffer with an ELISA washer (Biotek ELx50; BioTek Instruments, Winooski, VT, USA). Then, biotinylated detection antibody was added to bind with conjugates on the coated antibody. After washing off unbound conjugates, HRP-Streptavidin was added. After a third washing, TMB substrates were added to visualize the HRP enzymatic reaction. TMB was catalyzed by HRP to produce a blue-color product that turned yellow after adding a stop solution. The O.D. absorbance at 450 nm was read using a microplate reader (Biotek ELx800; BioTek Instruments, Winooski, VT, USA). The concentration of analyte in the sample was calculated by drawing a standard curve. The concentration of the target substance is proportional to the OD450 value.

#### 2.3.8. Statistical Analysis

To evaluate the normality of continuous variables, the Shapiro–Wilk test was administered. For variables that failed to meet normality requirements in more than two independent groups, Kruskal–Wallis and all pairwise multiple comparison tests were employed. In cases of two dependent, non-normally distributed measurements, Wilcoxon tests were conducted. Comparisons involving more than two dependent measures necessitated the employment of 2-way repeated-measure variance analysis, LSD multiple comparison tests for variables distributed normally, Friedman tests for non-normally distributed variables, and all pairwise multiple comparison tests.

## 3. Results

There was no significant difference between the mean ages of the participants (*p* > 0.05).

### 3.1. Participant Characteristics

Forty participants were included, allocated equally to the non-smoker and ex-smoker groups (*n* = 20 each), with each participant contributing a chemically activated and a diode-laser-activated hemi-arch (split-mouth design).

### 3.2. Clinical/Periodontal Findings

#### 3.2.1. Plaque Index (PI) Findings

The results for PI* values are shown in Table 1. In terms of non-smokers, no statistically significant difference was observed between the PI* measurements for three different time intervals (*p* > 0.05). On the other hand, in ex-smoker groups, significant differences were found between PI1*-PI3* and between PI2*-PI3* (*p* < 0.05). In the intergroup comparison, while no statistically significant difference was observed between all groups in PI1* and PI2* measurement (*p* > 0.05), a statistically significant difference was observed between non-smokers (Group 1/Group 2) and ex-smokers (Group 3/Group 4) in PI3* measurement (*p* < 0.05).

#### 3.2.2. Gingival Index (GI) Findings

The results for GI* values are shown in Table 1. In terms of non-smokers/ex-smokers, significant differences were found between GI1*-GI2* and GI2*-GI3* for both activation methods (*p* < 0.05). In the intergroup comparison, while no statistically significant difference was observed for the GI1* measurement (*p* > 0.05), significant differences were found between non-smokers (Group 1/Group 2) and ex-smokers (Group 3/Group 4) in both GI2* and GI3* measurements (*p* < 0.05).

#### 3.2.3. Bleeding Index (BI) Findings

The results for BI* values are shown in Table 1. In terms of non-smokers/ex-smokers, significant differences were found between BI1*-BI2* and BI2*-BI3* for both activation methods (*p* < 0.05). In the intergroup comparison, while no statistically significant differences were observed for both BI1* and BI2* measurements (*p* > 0.05), a significant difference was found between non-smokers (Group 1/Group 2) and ex-smokers (Group 3/Group 4) in BI3* measurements (*p* < 0.05).

### 3.3. GCF Cytokine Levels

#### 3.3.1. GCF TNF-α Findings

The mean levels of GCF TNF-α measurements are presented in Table 2. In terms of non-smokers, significant differences were found between TNF-α1 and TNF-α2, TNF-α1 and TNF-α3, TNF-α2 and TNF-α4, and TNF-α3 and TNF-α4 for the chemically activated treatment (*p* < 0.05). Nevertheless, no statistically significant difference was observed between TNF-α1 and TNF-α4 for non-smokers with laser activation and ex-smokers (*p* > 0.05). There were statistically significant differences between TNF-α2 and all other TNF-α levels (*p* < 0.05). Similarly, there were statistically significant differences between TNF-α3 and all other TNF-α levels (*p* < 0.05). In the intergroup comparison, statistically significant differences were found in TNF-α measurement levels between non-smokers (Group 1/Group 2) and ex-smokers (Group 3/Group 4) for TNF-α1, TNF-α3, and TNF-α4 measurements (*p* < 0.05). For the TNF-α2 measurements, no statistically significant difference was observed between the ex-smoker group with activation via a diode laser and other groups (*p* > 0.05).

#### 3.3.2. GCF IL-6 Findings

The mean levels of GCF IL-6 measurements are presented in Table 2. In terms of non-smokers, no statistically significant difference was observed between IL-61 and IL-64 (*p* > 0.05). There were statistically significant differences between IL-62 and all other IL-6 levels (*p* < 0.05). Similarly, there were statistically significant differences between IL-63 and all other IL-6 levels (*p* < 0.05). In terms of ex-smokers receiving chemical activation, there were statistically significant differences between IL-62 and all other IL-6 levels (*p* < 0.05). Similarly, there were statistically significant differences between IL-63 and all other IL-6 levels, except IL-64 (*p* < 0.05). On the other hand, in ex-smokers with diode laser activation, there were statistically significant differences between IL-62 and all other IL-6 levels (*p* < 0.05). In the intergroup comparison, statistically significant differences were found in IL-6 measurement levels between non-smokers (Group 1/Group 2) and ex-smokers (Group 3/Group 4) for IL-61, IL-63, and IL-64 measurements (*p* < 0.05). For the IL-62 measurements, no statistically significant difference was observed between the ex-smoker group with diode laser activation and other groups (*p* > 0.05).

### 3.4. Salivary Cytokine Levels

#### 3.4.1. Saliva TNF-α Findings

The mean level of salivary TNF-α measurements are presented in Table 3. In terms of non-smokers/ex-smokers, significant differences were found between TNF-α1 and TNF-α2, TNF-α1 and TNF-α3, TNF-α2 and TNF-α4, and TNF-α3 and TNF-α4 (*p* < 0.05). In the intergroup comparison, statistically significant differences were found in TNF-α measurement levels between ex-smokers and non-smokers for four different time intervals (*p* < 0.05).

#### 3.4.2. Saliva IL-6 Findings

The mean levels of salivary IL-6 measurements are presented in Table 3. In terms of non-smokers, no statistically significant difference was observed between IL-61 and IL-64 (*p* > 0.05). There were statistically significant differences between IL-62 and all other IL-6 levels (*p* < 0.05). Similarly, there were statistically significant differences between IL-63 and all other IL-6 levels (*p* < 0.05). In terms of ex-smokers, no statistically significant difference was observed between IL-61 and IL-64, and IL-62 and IL-63 (*p* > 0.05). There were statistically significant differences between IL-62 and all other IL-6 levels, except IL-63 (*p* < 0.05). In the intergroup comparison, statistically significant differences were found in IL-6 measurement levels between ex-smokers and non-smokers for four different time intervals (*p* < 0.05).

## 4. Discussion

In the literature, periodontal index values differ in smokers. The majority of evidence points towards smoking having a negative impact on oral health, including increased plaque accumulation, higher risk of periodontal disease, and potential alterations in the oral microbiome [24,25], while Kornman et al. [26] reported that smokers have less plaque. However, other studies have suggested that smoking does not have an impact on the amount of plaque present [27,28]. Based on our findings, it was observed that PI decreased due to bleaching. Despite the fact that PI was found to be higher in laser-activated bleaching cases in both groups, no statistically significant difference was noted between the PI values of the groups after bleaching. Furthermore, the bleaching treatments did not have any statistically significant impact on PI values. The values of GI and BI can vary among individuals, influenced by smoking habits. Research indicates that smokers exhibit higher values for both indices [27,29], while other studies reveal the opposite trend [30,31]. However, some studies do not show any significant difference in GI and BI values between smokers and non-smokers [32,33]. Our GI and BI measures increased in all groups in the post-treatment measurements, followed by a decrease after 2 weeks. In both groups, GI and BI values were found to be higher when the gel was activated by a laser.

The lower plaque index observed in the ex-smoker group should not be interpreted as direct evidence of reduced local inflammation, as PI is a measure of visible biofilm accumulation rather than an inflammatory marker. This finding may instead be hypothetically associated with a shift in biofilm microbial composition following smoking cessation, and/or with improved oral hygiene behavior commonly reported after cessation. We present this as a hypothesis-generating observation rather than as evidence of an anti-inflammatory effect, and it should be interpreted independently of the cytokine findings reported below.

The findings regarding BI indicated that it increased following the second session of whitening treatment for both ex-smokers and non-smokers. Previous research has posited that smoking may result in vasoconstriction of the gingival microcirculation, which could lead to decreased blood flow in the gingival tissues. Several studies suggest that smokers exhibit a lower incidence of gingival bleeding on probing than non-smokers. One possible reason for this difference is the vasoconstrictive effect of nicotine on the peripheral blood vessels; this effect is shown by nicotine as stimulation of adrenaline and noradrenaline on alpha-adrenergic receptors [34,35]. The reason for the decrease in bleeding in ex-smokers compared to non-smokers may be attributed to the impairment of the inflammatory response due to smoking, as well as the GI score. Considering these outcomes, the first null hypothesis was invalidated.

Saliva is an easily obtainable sample that is rich in biomarkers, making it an ideal choice for diagnosing and prognosing caries and evaluating the efficacy of treatment protocols, as well as detecting and monitoring periodontal diseases [36]. In our research, we have tested the levels of biomarkers such as IL-6 and TNF-α, which are indicators of local and systemic inflammation [37]. GCF comprises a diverse array of biochemical elements derived from sources such as serum, leukocytes, structural cells, bacteria, antibodies, cytokines, enzymes, and debris from damaged tissue. Due to these various components, GCF may possess diagnostic or prognostic potential as an indicator of changes in the periodontal tissues.

Cytokines are critical factors in the pathogenesis of inflammation and periodontal diseases, as they can exhibit either pro-inflammatory or anti-inflammatory properties. Despite their low concentration levels, they are highly effective. Additionally, they are involved in all phases of the immune response associated with periodontal diseases [36]. According to our understanding, limited research has been conducted to assess the impact of bleaching treatments on cytokine levels. There are no existing studies whose results can be directly compared with our findings. Surmelioglu et al. [38] investigated the local effects on DNA of various bleaching methods measuring serum, GCF, saliva samples, and 8-hydroxy-2′-deoxyguanosine (8-OHdG) levels. The study found that bleaching treatments did not affect serum and saliva 8-OHdG levels, but GCF 8-OHdG levels increased in the chemical and diode-laser-activated bleaching groups. Lima et al. [39] investigated the impact of 15% and 35% HP-containing in-office whitening treatments on cytokine levels in GCF from 25 individuals and found no substantial variation in cytokine levels across different time intervals. According to Benetti et al., IL-6 and IL-17 levels were observed to rise significantly on the second day after bleaching, a consequence of inflammation [40]. Another investigation on mice explored the consequences of bleaching therapy using 38% HP, which was assessed through immunohistochemical examination. This examination revealed strong staining for IL-1β, TNF-β, FGF-2, and glutathione peroxidase following bleaching. Based on these findings, it was inferred that bleaching procedures involving 38% HP might cause pulpal inflammation [41]. Bersezio et al. [42] reported a rise in the levels of IL-1β and RANK-L in the GCF following devital bleaching, a trend that persisted even after a 3-month follow-up period. Our study shows a temporary rise in TNF-α and IL-6 levels in saliva and GCF due to bleaching treatments, similar to previous research. Additionally, TNF-α and IL-6 levels in ex-smokers were found to be lower than those in non-smoker groups in all measurements.

Given that all ex-smokers in this cohort had discontinued smoking for more than 5 years, the lower TNF-α and IL-6 levels observed in this group cannot be attributed to acute nicotine-induced vasoconstriction, an effect that resolves within hours of exposure. We propose instead that these differences more plausibly reflect long-term consequences of prior tobacco exposure, including persistent immunomodulation and epigenetic alterations affecting local immune-cell reactivity (e.g., altered methylation of cytokine-gene promoters) that can outlast cessation for years, incomplete normalization of the oral microbiota composition following cessation, and/or chronic suppression of innate immune responsiveness and structural remodeling of the periodontal microvasculature and connective tissue established during the period of active smoking. These hypotheses, rather than an acute pharmacological mechanism, are proposed as more biologically plausible explanations for the cytokine differences observed in this long-term ex-smoker population; direct confirmation would require dedicated immunological, microbiological, and epigenetic analyses, which were beyond the scope of the present study. According to the current results, the second null hypothesis was disregarded.

### 4.1. Limitations

It is important to note that there are limitations to this study. Firstly, this study was conducted on healthy patients with smoking habits having a local effect, and did not include systemic effects. Secondly, this study only assessed the effect of cytokine levels and periodontal indices, and did not take into account other factors such as bleaching effectiveness, dentin hypersensitivity, or other cytokine markers. The split-mouth design compared two bleaching activation methods against one another but did not include a third, untreated control side; therefore, causal attribution of the observed biochemical and clinical changes specifically to the bleaching procedure, rather than to other concurrent factors, cannot be firmly established, and causal language has been avoided throughout. In the ex-smoker group, cumulative tobacco exposure (pack-years), time since smoking cessation, and objective indicators of residual nicotine exposure or microvascular effect (e.g., serum/salivary cotinine, gingival blood flow) were not assessed; therefore, residual effects of past smoking on periodontal tissue and inflammatory response may not have been fully captured. Smoking status was classified based on self-report at a structured screening interview; no biochemical verification (e.g., salivary or serum cotinine) was performed to objectively confirm non-smoking status or the absence of recent nicotine exposure, including from non-cigarette sources such as e-cigarettes; self-reported smoking history may be subject to recall or social-desirability bias. The data were analyzed using repeated ANOVA and t-test comparisons at each time point; this approach does not directly account for the within-cluster correlation arising from the split-mouth design, nor does it directly test the potential three-way interaction between smoking status, activation method, and time. As the study was conducted as part of a previously completed and approved thesis, it was not possible to convert the analysis plan to a mixed-effects model at this stage. This study also did not include an objective, instrument-based assessment of tooth color change, such as digital spectrophotometry or a standardized Vita shade guide comparison; color outcomes were therefore not independently verified beyond the methods described, and we cannot completely exclude the possibility that variation in bleaching-gel concentration or penetration into the dental and periodontal tissues contributed to, or confounded, the cytokine responses observed, independently of activation method or smoking status. Future studies may consider these factors when comparing the efficacy of bleaching agent effectiveness on patients with different smoking habits.

### 4.2. Future Research Directions

Future studies should build on these findings by incorporating a genuine untreated control arm, objective biochemical verification of smoking status (e.g., cotinine testing), and objective colorimetric or spectrophotometric assessment of bleaching outcomes. Dedicated mechanistic investigations—including epigenetic, microbiological, and immunological analyses—are also warranted to directly test the hypotheses proposed here regarding the long-term effects of prior smoking on periodontal and salivary cytokine profiles, using linear mixed-effects models with participant-level random effects to more robustly evaluate whether the temporal bleaching response differs according to smoking history and activation method. Finally, larger, multicenter studies with adequate power for higher-order interactions (smoking status × activation method × time) would help confirm and extend the present exploratory findings.

## 5. Conclusions

Within the limitations of this study, smoking history and bleaching activation method were both associated with the local (GCF) and whole-mouth (salivary) cytokine response to in-office dental bleaching, with evidence suggesting a differential temporal pattern between non-smokers and long-term ex-smokers. These findings support the relevance of considering smoking history when interpreting the inflammatory impact of bleaching activation methods, although confirmation in larger, controlled studies is warranted before specific clinical recommendations can be made.

## Figures and Tables

**Table 1:** PI, GI and BI measurement values.

**PI**	**Groups (Non-Smokers)**		**Groups (Ex-Smoker)**		
	**1 (Chemical)**	**2 (Laser)**	**3 (Chemical)**	**4 (Laser)**	***p***
PI_1_*	0.33 ± 0.32 ^aA^	0.34 ± 0.28 ^aA^	0.25 ± 0.17 ^aA^	0.24 ± 0.19 ^aA^	>0.005
PI_2_*	0.25 ± 0.24 ^aA^	0.31 ± 0.28 ^aA^	0.21 ± 0.21 ^aA^	0.22 ± 0.27 ^aA^	>0.005
PI_3_*	0.23 ± 0.28 ^aA^	0.27 ± 0.26 ^aA^	0.21 ± 0.33 ^bB^	0.21 ± 0.27 ^bB^	0.021
***p***	>0.005	>0.005	0.044	0.039	
**GI**	**Groups (Non-Smoker)**		**Groups (Ex-Smoker)**		
	**1 (Chemical)**	**2 (Laser)**	**3 (Chemical)**	**4 (Laser)**	
GI_1_*	0.51 ± 0.44 ^aA^	0.55 ± 0.4 ^aA^	0.37 ± 0.16 ^aA^	0.35 ± 0.18 ^aA^	>0.005
GI_2_*	0.73 ± 0.51 ^aB^	0.93 ± 0.59 ^aB^	0.51 ± 0.24 ^bB^	0.64 ± 0.28 ^bB^	<0.001
GI_3_*	0.53 ± 0.22 ^aA^	0.57 ± 0.32 ^aA^	0.38 ± 0.14 ^bA^	0.43 ± 0.20 ^bA^	0.038
***p***	0.003	<0.001	<0.001	0.024	
**BI**	**Groups (Non-Smoker)**		**Groups (Ex-Smoker)**		
	**1 (Chemical)**	**2 (Laser)**	**3 (Chemical)**	**4 (Laser)**	
BI_1_*	0.47 ± 0.26 ^aA^	0.46 ± 0.19 ^aA^	0.40 ± 0.24 ^aA^	0.40 ± 0.26 ^aA^	>0.005
BI_2_*	0.72 ± 0.25 ^aB^	0.83 ± 0.17 ^aB^	0.67 ± 0.24 ^aB^	0.69 ± 0.24 ^aB^	>0.005
BI_3_*	0.48 ± 0.21 ^aA^	0.49 ± 0.21 ^aA^	0.42 ± 0.24 ^bA^	0.41 ± 0.25 ^bA^	0.016
***p***	0.019	0.027	<0.001	<0.001	

Different letters in columns and rows indicate statistically significant differences. Lowercase letters represent statistical difference in the same row (intergroup), and uppercase letters represent statistical difference in the same column (intragroup). PI: plaque index; PI_1_: plaque index before bleaching treatment; PI_2_: plaque index after second session; PI_3_: plaque index after two weeks. GI: gingival index; GI_1_: gingival index before bleaching treatment; GI_2_: gingival index after second session; GI_3_: gingival index after two weeks. BI: bleeding index; BI_1_: bleeding index before bleaching treatment; BI_2_: bleeding index after second session; BI_3_: bleeding index after two weeks.

**Table 2:** TNF-α and IL-6 values in GCF samples.

**TNF-α**	**Groups (Non-Smoker)**		**Groups (Ex-Smoker)**	
	**1 (Chemical)**	**2 (Laser)**	**3 (Chemical)**	**4 (Laser)**
**TNF-α_1_**	19.16 ± 3.93 ^aA^	19.21 ± 3.85 ^aA^	12.51 ± 2.27 ^bA^	12.50 ± 2.72 ^bA^
**TNF-α_2_**	20.74 ± 4.77 ^aB^	22.52 ± 2.78 ^acB^	17.30 ± 4.01 ^bB^	18.90 ± 4.81 ^abB^
**TNF-α_3_**	20.35 ± 3.64 ^aB^	21.71 ± 3.39 ^aC^	15.67 ± 4.51 ^bC^	17.52 ± 5.95 ^bC^
**TNF-α_4_**	19.36 ± 4.38 ^aA^	20.26 ± 4.36 ^aA^	13.63 ± 4.31 ^bA^	15.19 ± 5.47 ^bA^
**IL-6**	**Groups (Non-Smoker)**		**Groups (Ex-Smoker)**	
	**1 (Chemical)**	**2 (Laser)**	**3 (Chemical)**	**4 (Laser)**
**IL-6_1_**	15.56 ± 3.54 ^aA^	15.55 ± 3.55 ^aA^	9.97 ± 3.19 ^bA^	9.99 ± 3.46 ^bA^
**IL-6_2_**	17.62 ± 3.50 ^aB^	18.02 ± 3.53 ^abB^	15.28 ± 3.74 ^acB^	16.39 ± 4.82 ^abcB^
**IL-6_3_**	16.17 ± 3.61 ^aC^	16.49 ± 3.45 ^aC^	11.24 ± 3.92 ^bC^	10.77 ± 3.43 ^bA^
**IL-6_4_**	15.72 ± 3.72 ^aA^	15.67 ± 3.58 ^aA^	10.10 ± 2.49 ^bAC^	10.05 ± 3.53 ^bA^

Different letters in columns and rows indicate statistically significant differences. Lowercase letters represent statistical difference in the same row (intergroup), and uppercase letters represent statistical difference in the same column (intragroup). TNF-α_1_: TNF-α values before bleaching treatment; TNF-α_2_: TNF-α values after first session; TNF-α_3_: TNF-α values after second session; TNF-α_4_: TNF-α values after two weeks. IL-6_1_: IL-6 values before bleaching treatment; IL-6_2_: IL-6 values after first session; IL-6_3_: IL-6 values after second session; IL-6_4_: IL-6 values after two weeks.

**Table 3:** TNF-α and IL-6 values in saliva samples reported solely as a non-smoker vs. ex-smoker comparison, not stratified by activation side, per the revised analytic strategy above.

**TNF-α**	**Group (Non-Smoker)**	**Group (Ex-Smoker)**
**TNF-α_1_**	19.86 ± 4.58 ^aA^	13.07 ± 4.01 ^bA^
**TNF-α_2_**	32.51 ± 5.18 ^aB^	24.27 ± 4.07 ^bB^
**TNF-α_3_**	31.56 ± 7.30 ^aB^	20.96 ± 5.96 ^bB^
**TNF-α_4_**	20.12 ± 5.37 ^aA^	13.22 ± 4.98 ^bA^
**IL-6**	**Group (Non-Smoker)**	**Group (Ex-Smoker)**
**IL-6_1_**	16.02 ± 4.94 ^aA^	9.22 ± 4.54 ^bA^
**IL-6_2_**	27.61 ± 5.42 ^aB^	16.73 ± 2.96 ^bB^
**IL-6_3_**	25.20 ± 4.71 ^aC^	14.84 ± 3.45 ^bB^
**IL-6_4_**	16.91 ± 4.12 ^aA^	9.32 ± 3.06 ^bA^

Different letters in columns and rows indicate statistically significant differences. Lowercase letters represent statistical difference in the same row (intergroup), and uppercase letters represent statistical difference in the same column (intragroup). TNF-α_1_: TNF-α values before bleaching treatment; TNF-α_2_: TNF-α values after first session; TNF-α_3_: TNF-α values after second session; TNF-α_4_: TNF-α values after two weeks. IL-6_1_: IL-6 values before bleaching treatment; IL-6_2_: IL-6 values after first session; IL-6_3_: IL-6 values after second session; IL-6_4_: IL-6 values after two weeks.

## Data Availability

The datasets used and/or analyzed during the current study are available from the corresponding author on reasonable request.

## References

[1] Irusa K., Alrahaem I.A., Ngoc C.N., Donovan T. (2022). Tooth whitening procedures: A narrative review. Dent. Rev..

[2] Komine C., Uchibori S., Tsudukibashi O., Tsujimoto Y. (2022). Application of reactive oxygen species in dental treatment. J. Pers. Med..

[3] Ionescu C., Kamal F.Z., Ciobica A., Halitchi G., Burlui V., Petroaie A.D. (2024). Oxidative stress in the pathogenesis of oral cancer. Biomedicines.

[4] Becker L.C., Cherian P.A., Bergfeld W.F., Belsito D.V., Hill R.A., Klaassen C.D., Liebler D.C., Marks J.G., Shank R.C., Slaga T.J. (2024). Safety assessment of hydrogen peroxide as used in cosmetics. Int. J. Toxicol..

[5] Aidos M., Marto C.M., Amaro I., Cernera M., Francisco I., Vale F., Marques-Ferreira M., Oliveiros B., Spagnuolo G., Carrilho E. (2024). Comparison of in-office and at-home bleaching techniques: An umbrella review of efficacy and post-operative sensitivity. Heliyon.

[6] Lau X.E., Liu X., Chua H., Wang W.J., Dias M., Choi J.J. (2023). Heat generated during dental treatments affecting intrapulpal temperature: A review. Clin. Oral Investig..

[7] Kiryk J., Kiryk S., Kensy J., Świenc W., Palka B., Zimoląg-Dydak M., Dobrzyński W., Matys J., Dobrzyński M. (2024). Effectiveness of laser-assisted teeth bleaching: A systematic review. Appl. Sci..

[8] Pournaghiazar F., Kimyai S., Motiei M. (2023). Effects of different light-assisted power bleaching techniques on the penetration of hydrogen peroxide into the pulp chamber. Photobiomodul. Photomed. Laser Surg..

[9] Tekce A.U., Yazici A.R. (2022). Clinical comparison of diode laser- and LED-activated tooth bleaching: 9-month follow-up. Lasers Med. Sci..

[10] Celik N., Askın S., Gul M.A., Seven N. (2017). The effect of restorative materials on cytokines in gingival crevicular fluid. Arch. Oral Biol..

[11] Chen C., Huang X., Zhu W., Ding C., Huang P., Li R. (2021). H_2_O_2_ gel bleaching induces cytotoxicity and pain conduction in dental pulp stem cells via intracellular reactive oxygen species on enamel/dentin disc. PLoS ONE.

[12] Llena C., Collado-González M., García-Bernal D., Oñate-Sánchez R.E., Martínez C.M., Moraleda J.M., Rodríguez-Lozano F.J., Forner L. (2019). Comparison of diffusion, cytotoxicity and tissue inflammatory reactions of four commercial bleaching products against human dental pulp stem cells. Sci. Rep..

[13] Ainamo J., Barmes D., Beagrie G., Cutress T., Martin J., Sardo-Infirri J. (1982). Development of the World Health Organization (WHO) community periodontal index of treatment needs (CPITN). Int. Dent. J..

[14] Singh A., Shrestha A., Bhagat T. (2021). Pain perception and dental anxiety during periodontal probing in patients visiting community oral health programme: A cross-sectional study. BMC Oral Health.

[15] Abrantes P.S., Xavier C.M., Melo A.M.D.S., Assunção I.V., Borges B.C.D. (2021). Efficacy, longevity, and bleaching sensitivity of carbamide and hydrogen peroxides for in-office bleaching: A 6-month randomized, double-blind, split-mouth clinical trial. Am. J. Dent..

[16] Kury M., Lins R.B.E., Resende B.A., Picolo M.Z.D., André C.B., Cavalli V. (2022). The influence of the renewal or the single application of the peroxide gel on the efficacy and tooth sensitivity outcomes of in-office bleaching: A systematic review and meta-analysis. J. Esthet. Restor. Dent..

[17] Anagnostaki E., Mylona V., Parker S., Cronshaw M., Grootveld M. (2023). Assessing the viability of laser-activated dental bleaching compared to conventional in-office bleaching methods: A systematic review of clinical and in vitro studies. Appl. Sci..

[18] Silness J., Löe H. (1964). Periodontal disease in pregnancy II. Correlation between oral hygiene and periodontal condition. Acta Odontol. Scand..

[19] Ainamo J., Bay I. (1975). Problems and proposals for recording gingivitis and plaque. Int. Dent. J..

[20] Löe H., Silness J. (1963). Periodontal disease in pregnancy I. Prevalence and severity. Acta Odontol. Scand..

[21] Enver A., Ozmeric N., Isler S.C., Toruner M., Fidan C., Demirci G., Elgun S., Silva A.P. (2022). Evaluation of periodontal status and cytokine levels in saliva and gingival crevicular fluid of patients with inflammatory bowel diseases. J. Periodontol..

[22] Çetin Özdemir E., Gündoğar H., Erciyas K., Üstün K., Şenyurt S.Z., Sezer U. (2021). Gingival crevicular fluid levels of cytokine, chemokine, and growth factors in patients with periodontitis or gingivitis and periodontally healthy subjects: A cross-sectional multiplex study. Cent. Eur. J. Immunol..

[23] Tou G.A.D.A., Diniz I.M.A., Ferreira M.V.L., Mesquita R.A., Yamauti M., Silva T.A., Macari S. (2022). Evaluation of periodontal parameters and gingival crevicular fluid cytokines in children with anterior open bite receiving passive orthodontic treatment with a spur. Korean J. Orthod..

[24] Al-Marzooq F., Al Kawas S., Rahman B., Shearston J.A., Saad H., Benzina D., Weitzman M. (2022). Supragingival microbiome alternations as a consequence of smoking different tobacco types and its relation to dental caries. Sci. Rep..

[25] La Rosa G.R., Di Stefano A., Gangi D., Emma R., Fala V., Amaliya A., Yilmaz H.G., Giudice R.L., Pacino S.A., Pedulla E. (2024). Dental plaque quantitation by light induced fluorescence technology in exclusive Electronic Nicotine Delivery Systems (ENDS) users. J. Dent..

[26] Kornman K.S. (2008). Mapping the pathogenesis of periodontitis: A new look. J. Periodontol..

[27] Axelsson P., Paulander J., Lindhe J. (1998). Relationship between smoking and dental status in 35-, 50-, 65-, and 75-year-old individuals. J. Clin. Periodontol..

[28] Kafle S., Gupta A., Shrestha E. (2026). Periodontal health status among cigarette smokers and nonsmokers in Chitwan District, Nepal: A comparative cross-sectional study. BioMed Res. Int..

[29] Malik A., Adnan M., Shahzad A., Anjum A., Ajmal J., Zohra S., Butt H. (2024). Prevalence of sensitivity and bleeding gums in smokers versus non-smokers. J. Health Rehabil. Res..

[30] Ehsan H. (2025). The influence of smoking on periodontal health: A case-control study in Afghanistan. J. Periodontol..

[31] Šutej I., Božić D., Peroš K., Plančak D. (2021). Cigarette smoking and its consequences on periodontal health in teenagers: A cross-sectional study. Cent. Eur. J. Public Health.

[32] Babayiğit O., Tastan Eroglu Z., Ozkan Sen D., Sarac F., Altun S., Ucan Yarkac F. (2026). Impact of smoking and periodontitis on salivary IL-39, IL-41, IL-1β, and TNF-α levels: A cross-sectional analysis. BMC Oral Health.

[33] Do H.T., Nguyen T.T., Vo T.L., Huynh N.C.N., Nguyen A.T.K. (2023). The influence of smoking on oral neutrophils and matrix metalloproteinase-8 in periodontitis patients before and after nonsurgical treatment. J. Oral Biol. Craniofac. Res..

[34] Al-Bayaty F.H., Baharuddin N., Abdulla M.A., Ali H.M., Arkilla M.B., ALBayaty M.F. (2013). The influence of cigarette smoking on gingival bleeding and serum concentrations of haptoglobin and alpha 1-antitrypsin. BioMed Res. Int..

[35] Silva H. (2021). Tobacco use and periodontal disease—The role of microvascular dysfunction. Biology.

[36] Arroyo E., Oliveira-Alves M.G., Chamorro-Petronacci C.M., Marichalar-Mendia X., Bravo-Lopez S.B., Blanco-Carrion J., Perez-Sayans M. (2023). Protein-based salivary biomarkers for the diagnosis of periodontal diseases: Systematic review and meta-analysis. J. Taibah Univ. Med. Sci..

[37] Relvas M., Mendes-Frias A., Gonçalves M., Salazar F., López-Jarana P., Silvestre R., Viana da Costa A. (2024). Salivary IL-1β, IL-6, and IL-10 are key biomarkers of periodontitis severity. Int. J. Mol. Sci..

[38] Sürmelioğlu D., Gündoğar H., Taysi S., Bağiş Y. (2021). Effect of different bleaching techniques on DNA damage biomarkers in serum, saliva, and GCF. Hum. Exp. Toxicol..

[39] Lima S.N., Ribeiro I.S., Grisotto M.A., Fernandes E.S., Hass V., de Jesus Tavarez R.R., Pinto S.C., Lima D.M., Loguercio A.D., Bandeca M.C. (2018). Evaluation of several clinical parameters after bleaching with hydrogen peroxide at different concentrations: A randomized clinical trial. J. Dent..

[40] Benetti F., Gomes-Filho J.E., Ferreira L.L., Sivieri-Araújo G., Ervolino E., Briso A.L., Cintra L.T. (2018). Concentration-dependent effect of bleaching agents on the immunolabelling of interleukin-6, interleukin-17 and CD5-positive cells in the dental pulp. Int. Endod. J..

[41] Silva-Costa R.S.G.d., Ribeiro A.E.d.L., Assunção I.V.d., Araújo R.F., Araújo A.A., Guerra G.C., Borges B.C. (2018). In-office tooth bleaching with 38% hydrogen peroxide promotes moderate/severe pulp inflammation and production of IL-1β, TNF-β, GPX, FGF-2 and osteocalcin in rats. J. Appl. Oral Sci..

[42] Bersezio C., Sánchez F., Estay J., Ledezma P., Vernal R., Garlet G., Oliveira O.B., Fernández E. (2020). Inflammatory markers IL-1β and RANK-L assessment after non-vital bleaching: A 3-month follow-up. J. Esthet. Restor. Dent..

